# Government regulation or market mechanism? A study on the collaborative innovation pathways of the emergency industry based on evolutionary game models

**DOI:** 10.1371/journal.pone.0322563

**Published:** 2025-05-12

**Authors:** Xinpu Zhang, Hongbo Li, Qi Kang, Lewei Chen

**Affiliations:** 1 School of Management, Jiangsu University, Zhenjiang, China; 2 China Institute for Agricultural Equipment Industrial Development, Jiangsu University, Zhenjiang, China; 3 School of Emergency Management, Jiangsu University, Zhenjiang, China; Tongji University School of Economics and Management, CHINA

## Abstract

Promoting collaborative innovation within the emergency industry has become a crucial task, which is of great significance for enhancing emergency response capabilities and ensuring public safety. On the basis of considering the economic attributes of the emergency industry and the uncertainty characteristics of market returns, this study constructs an evolutionary game model for collaborative innovation among emergency enterprises, universities and research institutions (UR), and government departments, which are the main entities considered. It analyzes the evolutionary process of the strategy choices and equilibrium states of different entities, thereby revealing the key factors and intrinsic mechanisms affecting collaborative innovation pathways. The study results indicate that the economic attributes of the emergency industry and the uncertainty of market returns are key factors that constrain the collaborative innovation and development of the emergency industry. The economic attributes of the emergency industry determine the applicable boundaries of government regulation and market mechanisms in the collaborative innovation process of the emergency industry. Due to the “marketization paradox” in the emergency industry with weak economic attributes, and the fact that emergency enterprises and UR are more likely to face the “prisoner’s dilemma” in the process of collaborative innovation, collaborative innovation via the government regulation-driven pathway is more feasible; In contrast, for the emergency industry with strong economic attributes, the government can effectively strengthen the market-oriented profit mechanism by enhancing public safety emergency awareness, thereby promoting collaborative innovation driven by market mechanisms in the emergency industry. Based on the analysis of the effects of different regulatory measures, it is found that government procurement is more effective than R&D subsidy policy. R&D subsidies are not only ineffective in avoiding the “prisoner’s dilemma” in the collaborative innovation process, but also have a “double-edged sword” effect. Excessive subsidy intensity can actually inhibit the enthusiasm of emergency enterprises and UR for collaborative innovation. When government departments adopt regulatory actions, focusing on balancing incentive and punitive measures, and emergency enterprises and UR focus on establishing a reasonable benefit distribution mechanism, which can more effectively improve the efficiency of collaborative innovation and form a good situation of win-win for all parties. The above findings provide certain decision-making references for the promotion of innovative development in the emergency industry.

## 1. Introduction

Natural disasters (e.g., earthquakes, floods, and typhoons) and human-made disasters (e.g., fires, traffic accidents, and chemical leaks) occur frequently worldwide, causing significant casualties and property losses to society, which threatens social stability and national security(Pelling, 2014) [1]. The 2023 Global Natural Disaster Assessment Report provides a detailed analysis, indicating that compared to previous years, the frequency of global natural disasters decreased by 3%, and the affected population declined by 53%. However, the death toll rose sharply by 73%, and direct economic losses increased by 32%, suggesting that the severity of individual disasters has worsened, leading to higher casualties and economic damage. Against this backdrop, the inadequacy of emergency response capabilities, particularly the lack of advanced high-end emergency equipment, has become a key factor contributing to the escalation of disaster-related losses and the inefficiency of rescue operations(Jia et al., 2022) [2]. Particularly, China is the country most severely affected by disasters worldwide. However, compared to developed countries, China’s emergency industry innovation capability is relatively lacking. Some emergency equipment often exhibits problems such as outdated technology, poor adaptability, and low efficiency when dealing with complex and changing disaster scenarios, making it difficult to meet the needs of modern emergency management(Shaw, 2017) [3]. For example, the lack of high-precision life detection equipment and large rescue machinery directly affects the timeliness and success rate of rescue in earthquake disasters. Therefore, in order to effectively respond to increasingly complex sudden disaster events, China urgently needs to further improve its emergency management system. As the core force supporting the operation of the emergency management system, the innovative development of the emergency industry is particularly important (Wang and Chen, 2022; Tang et al., 2022) [4,5].

The process of industrial innovation is often accompanied by high risks and costs, and relying solely on the strength of the enterprise itself to achieve innovation may face many challenges and limitations (Amnon, 2003) [6]. Industrial collaborative innovation, an effective way for countries around the world to reduce industrial innovation risks and costs (Hao et al., 2022; Luo et al., 2023) [7,8], refers to the joint technological research and development activities carried out by innovative entities such as enterprises, universities and research institutions (UR) through cooperation and resource complementarity (Veronica and Thomas, 2007; Rajalo and Maaja, 2017) [9,10]. However, the emergency industry is not a conventional economic sector. Constrained by its emergency-oriented nature, the external economic benefits brought by industrial innovation are not significant, resulting in low innovation enthusiasm among emergency enterprises, university and research institutions (UR), and the potential of industrial collaborative innovation has not been fully realized. Specifically, on the one hand, due to the special purposes of certain emergency products, especially the various emergency products and services provided during disaster rescue and emergency response stages, which often require government provision and cannot be profit oriented, this weakens the market economy attributes of such emergency industries to a certain extent, making it difficult to stimulate endogenous innovation in the emergency industry. On the other hand, the suddenness and complexity of disaster events determine that there is significant uncertainty in the technological application prospects of the emergency industry. For example, the frequency and intensity of natural disasters such as earthquakes and floods are difficult to predict, leading to a high degree of uncertainty in the research and market demand for emergency equipment. This further exacerbates the high-risk and high-cost characteristics of emergency industry innovation, and correspondingly suppresses the collaborative innovation enthusiasm of industry entities. The Chinese government attaches great importance to the above issues, and Five departments including the Ministry of Industry and Information Technology of China jointly issued the “Action Plan for the Development of Key Areas of Safety Emergency Equipment (2023-2025)”, which clearly proposes to promote Industry-University-Research (IUR) collaborative innovation through policy support. As the visible hand, the government’s interventions have a decisive impact on shaping both the direction of innovation and the market environment in the emergency industry. However, excessive reliance on government intervention may distort market mechanisms, leading to resource misallocation and inefficiency, thereby suppressing the motivation of enterprises and other innovation actors. Therefore, especially for emergency industries in the early stages of development, it is essential to further clarify the roles played by both government intervention and market mechanisms in the process of collaborative innovation in the emergency industry (Yu and Zheng, 2023) [11]. This leads to the following research questions: Under the dual influence of the economic attributes of the emergency industry and the uncertainty of market returns, what are the micro-level dynamics of collaborative innovation in the emergency industry? What are the applicable boundaries of government as the visible hand and the market as the invisible hand in collaborative innovation? If government regulation is needed, what measures would be most effective?

To address the above issues, this study attempts to introduce evolutionary game theory to systematically characterize the characteristics and evolutionary patterns of collaborative innovation in the emergency industry. Evolutionary game theory, as a theoretical tool for studying the strategy choices and evolution of bounded rational individuals in dynamic environments, is particularly suitable for this purpose As a theoretical tool for studying the strategic choices and evolution of bounded rational individuals in dynamic environments, evolutionary game theory can effectively simulate the strategy selection, behavioral adjustment, and the formation process of the final equilibrium state of multiple interacting agents (Smith, 1988; Traulsen, 2023) [12,13]. This provides a feasible approach for analyzing the paths of collaborative innovation in the emergency industry。Based on this, this paper constructs an evolutionary game model of collaborative innovation involving emergency enterprises, academic research institutions (UR), and the government, considering the economic attributes and market uncertainty characteristics of the emergency industry to analyze the key factors and underlying mechanisms that influence the collaborative innovation pathways in the emergency industry. This will help deepen the understanding of the intrinsic dynamics of collaborative innovation in the emergency industry and provide theoretical and practical implications for solving the real-world challenges of collaborative innovation in this field.

In terms of content arrangement, the second part of this paper is a literature review related to the emergency industry, industrial collaborative innovation, and the application of evolutionary game models; the third part presents the construction and evolutionarily stable strategy (ESS) analysis of the evolutionary game model; the fourth part presents the simulation analysis of the evolutionary game model; the fifth part is a summary of the research conclusions and insights of this study.

## 2. Literature review

### 2.1. Emergency industry innovation

The enhancement of national and regional emergency management capabilities must be supported by a robust emergency industry underpinned by a strong emergency technology system. In this regard, the academic community has conducted discussions on innovation in the emergency industry, including covering aspects such as the application of innovative technologies, the design of industrial policies and so on. Sandeep et al. studied the application of mobile communication technology in emergency disaster management, emphasizing its key role in helping disaster management personnel make decisions (2024) [14]. Martina et al.(2021) discussed the application of drone systems in emergency response scenarios, highlighting their efficiency and flexibility in disaster monitoring, material transportation, and search-and-rescue operations [15].In addition, digital twin technology (Enrico and Leonardo, 2024) [16]. IoT (Krichen,et al. 2024) [17]and the application of AI (Murthy et,al.,2025) [18] all contribute to the development of more effective and sustainable disaster management strategies. In summary, it is evident that the application of emergency innovation technologies is crucial for optimizing emergency safety management. In recent years, China has continuously increased its investment in emergency field research, and the industry as a whole has developed rapidly, with a wealth of research findings. In their studies, some scholars have pointed out that the development of China’s emergency industry should focus on emergency technology innovation (Guo Xiang, 2014) [19], as well as the construction of an emergency industry innovation system (Zhou, 2016; Xu et al., 2020) [20,21]. On the other hand, China’s industrial development is based on a combination of market mechanisms and government mechanisms, where the market mechanism plays a fundamental role and the government mechanism has a leading, regulatory, and supportive effect. Shen and Zhang (2024) discussed the transformation process of China’s emergency industry in depth, pointing out the cross-effect of government intervention, market dynamics, and social demand in the innovation and development process of the emergency industry; they suggested that the Chinese government should focus on social development needs and should guide the input of scientific and technological innovation in the emergency industry [22]. In addition, Yang and Jia (2023), based on the perspective of institutional grammar, used fuzzy set qualitative comparative analysis (fsQCA) to examine how policy design affects the innovation performance of China’s emergency industry; they found that policy design oriented by rules and standards could improve the innovation performance of China’s emergency industry, but that the lack of strategy-oriented policy design would lead to poor innovation performance [23].

### 2.2. Industrial collaborative innovation

The current scholarly research on industrial collaborative innovation mainly focuses on three aspects: connotation, impact effects, and mechanisms.

(1) The concept of industrial collaborative innovation can be analyzed from three dimensions: participants, process, and innovation network. In terms of participants, enterprises, universities, and research institutes are the core entities of industrial collaborative innovation. At the same time, other entities such as the government, financial institutions, and intermediary organizations may also be involved (Rajalo and Maaja, 2017) [10]. The government, as the policy maker and resource allocator, plays a crucial guiding and supporting role in collaborative innovation(Etzkowitz and Leydesdorff, 2000) [24]. From the process perspective, industrial collaborative innovation refers to a series of resource-sharing activities, such as technological cooperation, personnel exchange, information consultation, and achievement transformation, which are carried out through the division of labor and cooperation among related entities in the industrial chain (Jin et al., 2018) [25]. From the perspective of innovation networks, industry–university–research collaborative innovation is a collection of interactive relationships formed by multiple participating entities with networked and dynamic characteristics (Matteo et al., 2019) [26], and this networked structure facilitates the flow and diffusion of knowledge, thereby enhancing overall innovation efficiency (Powell et al., 1996) [27].
(2) Impact effects. Most studies affirm the positive impact of industrial collaborative cooperation on innovation performance. For example, Alessandra (2016) studied the impact of public-funded university collaborations with industry on corporate R&D efforts and found that participation in the project had a positive impact on R&D for each employee [28]. Using the triple helix theory, Liu et al. (2017) analyzed the positive effects of collaborative innovation carried out by governments, related enterprises, universities, and research institutions on product technology innovation and regional innovation [29]. Xie et al. (2023) explored the impact of two different types of collaborative innovation on innovation performance and found that supply chain (SC) collaborative innovation had a more significant effect on corporate innovation performance than innovation performance generated through industry–university–research cooperation (IUR) [30].
(3) The mechanisms of industrial collaborative innovation can be analyzed from three aspects: internal mechanisms, external mechanisms, and resource-sharing mechanisms. Zhou et al. (2013) proposed that internal incentives and profit drivers are the internal mechanisms of collaborative innovation, while government support is the external mechanism, and internal mechanisms emphasize mobilizing the motivation of all participating entities through benefit distribution and incentive mechanisms, while external mechanisms provide support for collaborative innovation through policy backing and resource investment [31]. Wang et al. (2021), using game theory, examined the optimal mechanism for profit distribution in the collaborative innovation process from a sustainability perspective, arguing that the optimal profit distribution for each enterprise should be directly proportional to its importance in the collaborative innovation project, which helps achieve the long-term stable development of collaborative innovation [32]. Yang et al. (2022) studied the impact of government subsidies on sustainable innovation in industry–university cooperation and found that subsidized industry–university collaborators generated more profit and greater social welfare [33]. In addition, knowledge and technology resource sharing is also a key mechanism for promoting collaborative innovation (Wu et al., 2019; Wang and Hu, 2020; Iqra et al., 2022) [34–36].

### 2.3. Evolutionary game models

John Maynard Smith and Price (1973) combined game theory with biological evolutionary theory to develop evolutionary game theory [37]. This theory breaks through the assumptions of perfect rationality and perfect information in traditional game theory, emphasizing the concept of bounded rationality, where participants typically cannot make optimal decisions at once but instead optimize their strategies through continuous trial and error and learning. It also emphasizes the long-term dynamic evolution process, analyzing how strategies spread, stabilize, or disappear within a group over time, thereby aligning more closely with the complexity and uncertainty of the real world [13]. Since then, evolutionary game theory has been applied to various disciplines, including economics, computer science, biology and management science(Arne and Nikoleta, 2023) [12]. As a result, Evolutionary game models have been widely applied in studying the innovation behaviors of industry players. By simulating the strategy adjustments and learning processes of participants in dynamic environments, they provide an effective analytical tool for studying the evolution of behaviors in complex systems (Mohammad, 2022; Zhang et al., 2023; Luo et al., 2023; Yuan and Li, 2024) [8,38–40]. For example, Zhang et al. (2023) constructed a tripartite evolutionary game model involving the government, enterprises, and the Energy Regulatory Service Center (ERSC) to explore the impact of government subsidies on the independent innovation of photovoltaic enterprises [38]. Luo et al. (2023) built a tripartite evolutionary game model aimed at agricultural enterprises, universities, and the government. From the perspective of innovation alliances, they examined the issue of low-carbon technology innovation in agriculture [8].

By reviewing relevant literature, it is found that existing studies primarily focus on the current state of innovation in the emergency industry, and the innovation mechanisms in this industry remain at a macro-theoretical level, lacking an in-depth exploration of the collaborative mechanisms between innovation entities from a micro perspective. Meanwhile, although there has been extensive discussion on industrial collaborative innovation mechanisms, existing research has not considered the specific characteristics of the emergency industry, particularly its economic attributes and market return uncertainties. Therefore, the findings of such studies may not effectively guide the practice of collaborative innovation in the emergency industry. Given this, it is both theoretically and practically necessary to conduct research on the micro-mechanisms of collaborative innovation in the emergency industry. Considering that the entities in the emergency industry (enterprises, academic and UR) are not completely rational in decision-making, but are influenced by information asymmetry and the uncertainties of the external environment, evolutionary game theory can better characterize such bounded rational behavior. Additionally, collaborative innovation in the emergency industry is a long-term, dynamic process involving strategic interactions and behavioral adjustments among multiple entities. Using evolutionary game theory can reveal the dynamic evolution of strategies among micro-level entities. Therefore, based on the characteristics of the emergency industry, this study constructs a multi-agent evolutionary game model involving the government, emergency enterprises, and UR to explore the pathways of collaborative innovation in the emergency industry. The innovations and marginal contributions of this study are as follows:

(1) This study explores the micro-mechanisms of collaborative innovation in the emergency industry based on evolutionary game theory. By analyzing the strategy choices and interactions between the government, emergency enterprises, and UR in the collaborative innovation process, it fills the gap in existing research at the micro level and expands the application of evolutionary game theory.
(2) This study breaks through the traditional analytical framework of industrial collaborative innovation by incorporating the economic attributes and market return uncertainties of the emergency industry into the research scope. It investigates the unique collaborative innovation pathways in the emergency industry. The findings not only reveal the intrinsic patterns of collaborative innovation in the emergency industry but also further enrich the theoretical understanding of industrial collaborative innovation.
(3) This study analyzes the collaborative innovation pathways of the emergency industry under the dual influence of government regulation and market mechanisms, clarifying the applicable boundaries of market mechanisms and government regulation in the collaborative innovation process. This study is of great practical significance, providing a reference for the government to develop targeted policies and contributing to the promotion of collaborative innovation in the emergency industry.

## 3. Model construction and analysis

### 3.1. Problem description and model assumptions

In the collaborative innovation development of the emergency industry, three main entities are involved: emergency enterprises (E), university and research institutions (UR), and government departments (G). Emergency enterprises and UR each have their focuses but are interdependent in the process of collaborative innovation in the emergency industry; they promote the rapid development and technological advancement of the emergency industry through close cooperation and joint efforts. Emergency enterprises need to closely monitor market demands, clarify the actual needs for emergency equipment and services through market research and customer feedback, and provide a clear direction for research and development. Furthermore, they are responsible for transforming research and development results into actual products for market promotion and application, thereby gaining economic benefits from innovation investment. UR focus on basic research related to the emergency industry, including the exploration of disaster early warning models, emergency communication technologies, new materials, and other cutting-edge technologies. In addition, they also undertake the task of training professional talents for the emergency industry. Emergency enterprises (E) and UR establish close industry–university–research cooperation through the construction of joint research and development platforms and the joint declaration of projects, actively promoting the sharing of technology, talents, and resources; they carry out technology research and development and achievement transformation through a (cost-)sharing approach, thereby achieving collaborative innovation and development in the emergency industry. Government departments (G) provide financial subsidies to support industry–university–research collaborative research and development projects, or they provide market application space for collaborative innovation results through government procurement methods. In addition, government departments also intervene in the market demand side of emergency products and services, including promoting emergency safety knowledge and culture, establishing emergency safety knowledge popularization and education bases, and the promotion of emergency safety knowledge, to enhance public emergency awareness and accelerate the promotion and application of emergency innovation products.

**Fig 1:**
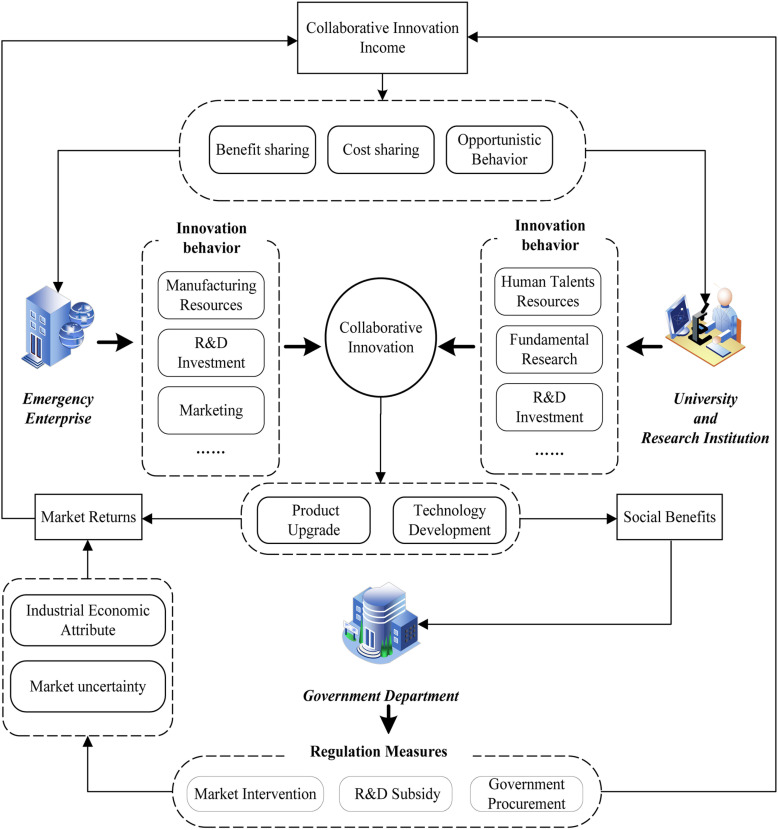
Theoretical model of collaborative innovation in emergency industry.

Based on the above, a theoretical model of collaborative innovation in the emergency industry can be constructed, as shown in Fig 1.

Fig 1 shows the relationships among the emergency enterprise (E), UR (U), and government department (G) in the process of collaborative innovation.

**Assumption 1**: All the entities have bounded rationality, and their behavioral strategies need to be repeatedly learned and adjusted to reach an optimal state. Emergency enterprises and UR have two behavioral strategies, namely Active Innovation and Passive Innovation; the government department’s strategy set is Regulation and Laissez-faire. The probabilities of emergency enterprises and UR choosing active innovation are  x  and y, respectively, and the probabilities of choosing passive innovation are 1−x and 1−y, respectively; the probabilities of the government department choosing regulation and laissez-faire are z and 1−z, respectively. Here, x,y,z∈[0,1].

**Assumption 2**: Regardless of whether emergency enterprises and UR choose active innovation, their basic benefits under normal operations are $R_{1}$ and $R_{2}$, respectively; only when both parties choose active innovation can technological progress and product upgrades be successfully achieved, with the resulting market economic benefits being ΔR.Specifically, we incorporates the following characteristic factors of the emergency industry into the assumption of collaborative innovation benefits: First, the size of the collaborative innovation benefits is constrained by the economic attributes of the industry, the higher this economic benefit, the stronger the economic attributes of the industry (Yu and Zheng, 2023) [11]. Some emergency products (such as escape devices for earthquakes, aviation rescue equipment, etc.) have specialized uses, limited market sizes, small demand potential, and restricted growth space. Therefore, collaborative innovation in such emergency industries is unlikely to generate significant economic benefits under market mechanisms, reflecting weak economic attributes. Second, the occurrence of sudden safety events is highly uncertain. Emergency products often exhibit the nature of ‘needed urgently when required, but unnecessary when not required’, which results in considerable uncertainty in the market benefits of emergency products. On the other hand, public awareness of emergency safety triggers preventive behaviors when facing potential risks. This means that even if a disaster does not necessarily occur, individuals will still choose to purchase and stock emergency products in preparation for potential unforeseen dangers (Ehrlich and Becker, 1972) [41]. Clearly, such behavior reduces the uncertainty in market demand and benefits to some extent. To align the model with the specific characteristics of the emergency industry, Referencing the research of Liu et al. (2024) [42], the collaborative innovation benefits is assumed to be ΔR=r−(1−evarepsilon, where r represents the economic attributes of the emergency industry, e (0<e<1) represents public emergency safety awareness and ε is a random variable representing innovation benefits, which follows a uniform distribution over the interval [0,u] (Yi et al.,2023; Jing et al., 2020) [43,44]. Clearly, the larger the interval length u, the broader the range of values for the random variable ε, meaning the higher the degree of uncertainty. Consequently, the expected benefits from collaborative innovation for industry players will be lower. For the convenience of model description, u is defined as the degree of uncertainty in the emergency industry’s benefits. In order to ensure that the additional benefits from collaborative innovation E(ΔR) is positive, it is assumed that u<2r.

**Assumption 3**: In the process of collaborative innovation in the emergency industry, emergency enterprises and UR actively promote the sharing of technology, talent, and resources and carry out technology research, development, and the transformation of achievements through a shared benefits and cost-sharing approach. Therefore, it is assumed that the total cost is $C_{I}$, with the emergency enterprise bearing a proportion of b and UR bearing a proportion of 1−b; the government provides financial support, and the distribution ratios of the innovation benefits are a and 1−a, respectively, with the emergency enterprise receiving a proportion of a and UR receiving a proportion of 1−a. Due to the knowledge spillovers present in the collaborative innovation process, under conditions of information asymmetry, one party may engage in opportunistic behavior to serve its own interests by choosing passive innovation [45] (Qi and Li, 2022), thus obtaining spillover benefits. The spillover benefits for the emergency enterprise and UR are $S_{1}$ and $S_{2}$, respectively. At the same time, the party choosing active innovation, in addition to the sunk costs of innovation investment, will also suffer additional economic losses. To simplify the model, it is assumed that the total loss for the party choosing active innovation is $C_{L}$. Because the party choosing passive innovation breaches the cooperation agreement, it must pay a penalty L when both parties choose passive innovation, additional losses and penalty payments are not considered (Guo et al., 2024) [46].

**Assumption 4**: When implementing regulatory measures, government departments will provide R&D subsidies to collaborative innovation projects involving emergency enterprises and UR, with the subsidy intensity denoted as W (W≥0). On the other hand, government departments will also create market application opportunities for collaborative innovation outcomes through centralized procurement, with the procurement intensity denoted as K (K≥0)*.* it should be noted that when implementing two types of incentive-based regulatory measures mentioned above, the government will supervise the behavior of emergency enterprises and UR to prevent strategic behaviors such as falsification, which will incur corresponding regulatory costs, denoted as $C_{F}$. Punishment will be imposed accordingly, resulting in losses for any party that chooses passive innovation*.* The punishment intensity is denoted as F (F≥0). In addition, the government enhances public awareness of emergency safety through the promotion of emergency safety knowledge culture, the construction of emergency safety knowledge popularization and education bases and other means. In addition, when the government does not take any measures to enhance public emergency awareness (e.g., promoting emergency safety knowledge and culture, establishing emergency safety knowledge popularization and education bases, etc.), $e=e_{0}$. When such measures are implemented, $e=e_{1}\;$($0<e_{0}<e_{1}<1$). Obviously, the government’s implementation of relevant measures will incur corresponding costs. Moreover, as public awareness of safety increases, it becomes more challenging for the government to further raise this awareness, leading to higher associated costs. Therefore, it is assumed that the cost, denoted as $C_{E}$, is a quadratic function of emergency safety awareness, specifically $C_{E}=\mu {(e}_{1}^{2}-e_{0}^{2})$,where μ is the cost coefficient. In summary, under government regulation, when the emergency industry achieves high-quality development due to collaborative innovation, it will generate socio-economic benefits, enhance government reputation, and receive rewards from higher-level governments. The total of these benefits is recorded as M (Zhang et al., 2023) [38].

### 3.2. Model construction

Under the assumptions mentioned above, a payoff matrix for the game among the three parties involved in the collaborative innovation process is constructed, as shown in Table 1.

**Table 1:** Payoff matrix.

Players				Government Departments (G)	
				Regulation z	Laissez-faire 1−z
**Emergency Enterprises (E)**	Active Innovation x	**University and Research Institutions** **(U)**	Active Innovation y	$R_{1}-bC_{I}+a\left(r-\left(1-e_{1}\right)E(\varepsilon )+K+W\right)$; ; $M-C_{F}-C_{E}-K-W$;	$R_{1}-bC_{I}+a\left(r-\left(1-e_{0}\right)E(\varepsilon )\right)$; ; 0;
			Passive Innovation 1−y	$R_{1}-C_{L}+L+\alpha W$; $R_{2}+S_{2}-L-F+(1-\alpha )W$; $F-C_{F}-C_{E}-W$;	$R_{1}-C_{L}+L$; $R_{2}+S_{2}-L$; 0;
	Passive Innovation 1−x	**University and Research Institutions** **(U)**	Active Innovation y	$R_{1}+S_{1}-L-F+\alpha W$; $R_{2}-C_{L}+L+(1-\alpha )W$; $F-C_{F}-C_{E}-W$	$R_{1}+S_{1}-L$; $R_{2}-C_{L}+L$; 0;
			Passive Innovation 1−y	$R_{1}-F+\alpha W$; $R_{2}-F+(1-\alpha )W$; $2F-C_{F}-C_{E}-W$;	$R_{1}$; $R_{2}$; 0;

Table 1 shows the payoff matrix for the game among the emergency enterprises (E), UR (U), and government departments (G).

Based on the payoff matrix, the following can be derived

(1) Replicator Dynamic Equation for the Strategy Choice of the emergency enterprises

$$\begin{array}{cc} U_{E1} & =yz\left(R_{1}-bC_{I}+a\left(r-\left(1-e_{1}\right)E(\varepsilon )+K+W\right)\right)+(1-y)z\left(R_{1}-C_{L}+L+aW\right)\\ & +y(1-z)\left(R_{1}-bC_{I}+a\left(r-\left(1-e_{0}\right)E(\varepsilon )\right)\right)+(1-y)(1-z)\left(R_{1}-C_{L}+L\right) \end{array}$$

$$U_{E0}=yz\left(R_{1}+S_{1}-L-F+aW\right)+(1-y)z\left(R_{1}-F+aW\right)+y(1-z)\left(R_{1}+S_{1}-L\right)+(1-y)(1-z)R_{1}$$

$$U_{E}=xU_{E1}+(1-x)U_{E0}$$

where

$U_{E1}$ is the expected payoff for the emergency enterprise when it chooses active innovation;

$U_{E0}\;$ is the expected payoff for the emergency enterprise when it chooses passive innovation;

$U_{E}$ is the average payoff for the emergency enterprises.

Thus, the replicator dynamic equation for the strategy choice of the emergency enterprises is

(2) Replicator Dynamic Equation for the Strategy Choice of UR

$$\begin{array}{cc} U_{U1} & =xz\left(R_{2}-(1-b)C_{I}+(1-a)\left(r-\left(1-e_{1}\right)E(\varepsilon )+K+W\right)\right)+(1-x)z\left(R_{2}-C_{L}+L+(1-a)W\right)\\ & +x(1-z)\left(R_{2}-(1-b)C_{I}+(1-a)\left(r-\left(1-e_{0}\right)E(\varepsilon )\right)\right)+(1-y)(1-z)\left(R_{2}-C_{L}+L\right) \end{array}$$

$$U_{U0}=yz\left(R_{2}+S_{2}-L-F+(1-a)W\right)+(1-y)z\left(R_{2}-F+(1-a)W\right)+y(1-z)\left(R_{2}+S_{2}-L\right)+(1-y)(1-z)R_{2}$$

$$U_{U}=yU_{U1}+(1-y)U_{U0}$$

where

$U_{U1}$ is the expected payoff for UR when it chooses active innovation;

$U_{U0}$ is the expected payoff for UR when it chooses passive innovation;

$U_{U}$ is the average payoff for UR.

Thus, the replicator dynamic equation for the strategy choice of UR is


$$F(y)=y(1-y)\left(L-C_{L}+zF+x\left(\begin{array}{c} C_{L}-S_{2}-(1-b)C_{I}+(1-a)\left(r-\frac{u\left(1-e_{0}\right)}{2}\right)\\ +z(1-a)(K+\frac{u\left(e_{1}-e_{0}\right)}{2}) \end{array}\right)\right)$$

(3) Replicator Dynamic Equation for the Strategy Choice of Government Department

$$\begin{array}{cc} U_{G1} & =xy\left(M_{1}-C_{F}-C_{E}-K\right)+(1-x)y\left(M+F-C_{F}-C_{E}-W\right)+x(1-y)\left(M+F-C_{F}-C_{E}-W\right)\\ & +(1-x)(1-y)\left(2F-C_{F}-C_{E}-W\right) \end{array}$$

$$U_{G0}=0$$

$$U_{G}=zU_{G1}+(1-z)U_{G0}$$

where

$U_{G1}$ is the expected payoff for government department when it chooses regulation;

$U_{G0}$ is the expected payoff for government department when it chooses laissez-faire;

$U_{G}$ is the average payoff for government departments.

Thus, the replicator dynamic equation for the strategy choice of government department is


$$F(z)=z(1-z)\left((1-x)F+(1-y)F-C_{F}-C_{E}-W-xyK+xyM\right)$$

### 3.3. Model solution and analysis

The replicator dynamic equations for the emergency enterprise, UR, and government department are set to 0, i.e., F(x)=0,F(y)=0,F(z)=0. Solving these equations together can yield eight pure strategy evolutionary equilibrium points, which are (0,0,0),(1,0,0),(0,1,0),(0,0,1),(1,1,0),(0,1,1),(1,0,1)and (1,1,1). By taking the partial derivatives of the replicator dynamic equations F(x),F(y),and F(z) with respect to x, yand z, the Jacobian matrix can be obtained:


$$J=[\begin{array}{ccc} \frac{\partial F(x)}{\partial x} & \frac{\partial F(x)}{\partial y} & \frac{\partial F(x)}{\partial z}\\ \frac{\partial F(y)}{\partial x} & \frac{\partial F(y)}{\partial y} & \frac{\partial F(y)}{\partial z}\\ \frac{\partial F(z)}{\partial x} & \frac{\partial F(z)}{\partial y} & \frac{\partial F(z)}{\partial z} \end{array}]$$

Table 2 shows the eigenvalues of the Jacobian matrix corresponding to each equilibrium point.

**Table 2:** Eigenvalues of the Jacobian matrix for each equilibrium point.

Equilibrium Point	Eigenvalue $\lambda_{1}$	Eigenvalue $\lambda_{2}$	Eigenvalue $\lambda_{3}$
(0,0,0)	$L-C_{L}$	$L-C_{L}$	$2F-C_{F}-C_{E}-W$
(1,0,0)	$C_{L}-L$		$F-C_{F}-C_{E}-W$
(0,1,0)	$L-S_{1}-bC_{I}+a\left(r-\frac{u\left(1-e_{0}\right)}{2}\right)$	$C_{L}-L$	$F-C_{F}-C_{E}-W$
(0,0,1)	$F+L-C_{L}$	$F+L-C_{L}$	$-2F+C_{F}+C_{E}+W$
(1,1,0)	$S_{1}-L+bC_{I}-a\left(r-\frac{u\left(1-e_{0}\right)}{2}\right)$		$M-C_{F}-C_{E}-W-K$
(0,1,1)	$F+L-S_{1}-bC_{I}+a(K+r-\frac{u\left(1-e_{1}\right)}{2})$	$C_{L}-F-L$	$C_{F}+C_{E}+W-F$
(1,0,1)	$C_{L}-F-L$	$F+L-S_{2}-(1-b)C_{I}+(1-a)(K+r-\frac{u\left(1-e_{1}\right)}{2})$	$C_{F}+C_{E}+W-F$
(1,1,1)	$S_{1}-F-L+bC_{I}-a(K+r-\frac{u\left(1-e_{1}\right)}{2})$	$S_{2}-F-L+(1-b)C_{I}-(1-a)(K+r-\frac{u\left(1-e_{1}\right)}{2})$	$C_{F}+C_{E}+W+K-M$

An equilibrium point is considered stable (an evolutionarily stable strategy, ESS) if Det(J)>0 and Tr(J)<0 (Friedman, 1991) [47]. Fluctuations in the stability of equilibrium points due to different parameter values lead to corresponding changes in the evolutionary path of the evolutionary game model. From this, the following proposition can be derived.

**Proposition 1** (1) When $L<C_{L}$ and $F<\frac{W+C_{E}+C_{F}}{2}$, the strategy (0,0,0) is evolutionarily stable strategy (ESS).

(2) When $F+L<C_{L}$ and $F>\frac{W+C_{E}+C_{F}}{2}$, the strategy (0,0,1) is evolutionarily stable strategy (ESS).

Proposition 1 indicates that, whether under market mechanisms or government regulation, emergency enterprises and UR may lose their enthusiasm for innovation investment, leading the collaborative innovation in the emergency industry to fall into a “negative stagnation” of low-end lock-in. The reason lies in the lack of effective supervision and penalty mechanisms during the collaborative innovation process, which fosters passive innovation behavior. In such cases, active investment by either party in the collaborative innovation process not only fails to increase their own returns but may also result in losses due to the opportunistic behavior of the other party. Therefore, passive innovation becomes the dominant strategy for emergency enterprises and UR. For government departments, if they excessively rely on incentive measures such as innovation cost subsidies and emergency product procurement to address the issue of low innovation economic efficiency caused by the weak economic attributes of the emergency industry, they may ultimately abandon regulation due to excessive fiscal cost pressures. Moreover, even if the government adopts a lower regulatory intensity to avoid high regulatory costs, it will neither help suppress opportunistic behavior in the collaborative innovation of the emergency industry nor change the passive innovation behavior of the industry entities, resulting in a situation of “regulatory failure.”

In summary, when the opportunistic behavior of internal entities in the emergency industry lacks constraints, coupled with insufficient or absent government regulation, collaborative innovation in the emergency industry will fall into a “stalemate” of negative stagnation. This situation often occurs during the initial stage of collaborative innovation development in the emergency industry. In this embryonic stage, the cooperative foundation between emergency enterprises and UR is relatively weak, lacking long-term and stable trust relationships. The absence of a trust mechanism increases the costs of cooperation in collaborative innovation, making it easier for opportunistic behavior to arise, thereby reducing the motivation for active participation by all parties. At the same time, the government’s initial interventions to promote collaborative innovation in the emergency industry may prove ineffective due to poorly designed policies or inadequate implementation.

**Proposition 2** (1) When $L>C_{L}$ and $F<W+C_{E}+C_{F}$, the strategy (1,0,0) is evolutionarily stable strategy (ESS) if ; the strategy (0,1,0) is evolutionarily stable strategy (ESS) if$a\left(r-\frac{\left(1-e_{0}\right)u}{2}\right)-{bC}_{I}<S_{1}-L$.

(2) When $F+L>C_{L}$ and $W+C_{E}+C_{F}<F$, the strategy (1,0,1) is evolutionarily stable strategy (ESS)if ; the strategy (0,1,1) is evolutionarily stable strategy (ESS) if $a\left(r-\frac{\left(1-e_{1}\right)u}{2}\right)-{bC}_{I}+F<S_{1}-L$.

Proposition 2 indicates that, whether under market mechanisms or government regulation, even if the opportunistic behavior of internal entities in the emergency industry is effectively constrained, there may still be situations where only one party (either emergency enterprises or universities/research institutions) chooses to actively innovate, resulting in “unilateral enthusiasm” in the collaborative innovation of the emergency industry. The reason lies in the fact that, during the collaborative innovation process between emergency enterprises and UR, although the penalty mechanisms for passive innovation behavior are strengthened, the weak economic attributes of the emergency industry make it difficult to establish an effective market-driven revenue mechanism. In other words, active innovation still struggles to generate substantial returns, while entities adopting passive innovation strategies can still obtain certain speculative gains. Additionally, even if the government alleviates the issue of insufficient external returns for emergency industry innovation through measures such as procurement of emergency innovation products, subsidies for innovation costs, and emergency safety awareness campaigns, if internal conflicts such as unbalanced benefit distribution and unreasonable cost-sharing among industry entities remain unresolved, the party receiving lower returns is more likely to adopt short-term passive innovation strategies. Consequently, the government may abandon regulation due to its limited effectiveness and high costs. It is also worth noting that if the government adopts low-intensity regulation (e.g., loose supervision mechanisms or ambiguous policy guidance) to alleviate fiscal pressure, the emergency industry will not escape the “unilateral enthusiasm” dilemma in collaborative innovation. Surprisingly, the government will not abandon regulation in such cases. This implies that even if the “unilateral enthusiasm” dilemma in the collaborative innovation of the emergency industry cannot be effectively resolved, the government will still repeatedly invest resources, which clearly leads to the waste of administrative resources.

From Propositions 1 and 2, it can be concluded that achieving collaborative innovation in the emergency industry faces two main challenges: First, the weak economic attributes of the emergency industry result in limited market-driven returns, which is the root cause of insufficient external incentives for collaborative innovation. Second, there are inherent conflicts among internal industry entities (emergency enterprises and UR), including the emergence of opportunistic behavior, imbalances in benefit distribution mechanisms, and unreasonable cost-sharing rules. These issues further undermine the stability and sustainability of collaborative innovation. Therefore, whether and how the government can implement effective regulation is key to breaking the “low-enthusiasm equilibrium trap” in the collaborative innovation of the emergency industry. The following propositions will further explore this issue.

**Proposition 3** (1) When $a\left(r-\frac{\left(1-e_{0}\right)u}{2}\right)-{bC}_{I}>S_{1}-L$, and $M<W+C_{E}+C_{F}+K$, the strategy (1,1,0) is evolutionarily stable strategy (ESS).

(2) When $a\left(r-\frac{\left(1-e_{1}\right)u}{2}\right)-{bC}_{I}+F>S_{1}-L$, and $M>W+C_{E}+C_{F}+K$, the strategy (1,1,1) is evolutionarily stable strategy (ESS).

Proposition 3 indicates that the feasible pathways for collaborative innovation in the emergency industry. Specifically, for emergency industries with weak economic attributes, the government regulation-driven pathway for collaborative innovation is significantly viable. This is because such emergency industries often face issues such as insufficient market demand, unstable investment returns, and high risks in technological innovation, making it difficult for market mechanisms to effectively drive their innovation and development. To address this, the government can promote collaborative innovation in the emergency industry through the following two measures: On one hand, the government can enhance public and institutional awareness and demand for emergency products and services, while utilizing government procurement channels to establish a regular mechanism for the storage and renewal of emergency supplies, thereby creating stable market demand. This measure can effectively increase the external returns of collaborative innovation in the emergency industry, reduce the negative impacts of market uncertainty, and provide a foundation for sustainable development. On the other hand, the government can directly reduce the R&D costs of enterprises through policy tools such as innovation subsidies, alleviating the financial pressure on enterprises during the initial stages of technological development. Such support can incentivize enterprises to engage in high-risk, high-investment innovation activities, driving technological advancement and industrial upgrading in the emergency industry.

Additionally, it has been found that if the emergency industry has strong economic attributes, even if the government does not implement regulatory measures, emergency enterprises and UR may still choose to actively innovate during the collaborative innovation process. This indicates that the market mechanism-driven pathway for collaborative innovation is feasible. However, it is important to note that for emergency industries with strong economic attributes, although their inherent market mechanisms have the potential to drive innovation, this potential has not yet been fully realized at the current stage. The main reason lies in the relatively weak emergency awareness among the general public, leading to a lack of sensitivity and proactiveness in the demand for emergency products and services. Taking household emergency products as an example, despite their enormous market potential, the general public’s awareness of emergency safety is generally low, and their understanding of emergency products and services is insufficient. As a result, such products are often perceived as “non-essential” or “low-frequency demand”, leading to weak consumer perception of the upgraded and innovative features of emergency technologies or products. This consumer psychology results in fragmented and short-term market demand, making it difficult to form stable market benefits and thereby constraining the innovation efficiency of the emergency industry. Therefore, for emergency industries with strong economic attributes, the government needs to carefully determine the timing for introducing public resource elements.

The above conclusions provide the following managerial implications: When promoting the development of the emergency industry, the government should formulate differentiated policies based on the economic attributes of different sub-sectors and implement categorized management of emergency products. For emergency sectors with weak economic attributes (e.g., public safety, disaster rescue, etc.), where the social benefits are significant but market-driven incentives are insufficient, the government should remain the main driver, ensuring development through fiscal support, government procurement, and other means, while using market mechanisms as a supplementary force. For emergency industries with strong economic attributes (e.g., household emergency equipment, emergency technical services, etc.), initial development should focus on enhancing public emergency awareness through multi-level interventions (e.g., promoting emergency knowledge through public awareness campaigns, community training, and school education), thereby guiding market demand towards scaling up, strengthening market-driven mechanisms, and improving the external economic benefits of innovation in emergency products, services, and technologies. In the mid-stage of development, both the government and the market should collaborate to further enhance the innovation motivation of the key players in the emergency industry. By the later stages of industrial development, as market mechanisms become more mature, the external economic benefits of innovation in the emergency industry will significantly increase, and industry players will possess intrinsic innovation drivers. At this point, the government should gradually withdraw from market regulation and promote the market-oriented development of emergency industry innovation, achieving self-organization and sustainable development of the industry.

**Proposition 4** (1) When the strategy (1,1,0) is evolutionarily stable strategy (ESS), the prisoner’s dilemma arises if $a\left(r-\frac{\left(1-e_{0}\right)u}{2}\right)<{bC}_{I}$ and .

(2) When the strategy (1,1,1) is evolutionarily stable strategy (ESS), the prisoner’s dilemma arises if $a\left(r-\frac{\left(1-e_{1}\right)u}{2}+K\right)+F<{bC}_{I}$ and 

Proposition 4 indicates that the potential ‘Prisoner’s Dilemma’ that may arise during the process of collaborative innovation in the emergency industry. Specifically, when the economic attributes of emergency products are weak, or when the government’s penalties for passive innovation behavior under R&D subsidy policies, the procurement of emergency innovation products, and the intensity of emergency safety awareness campaigns are relatively low, emergency enterprises and academic research institutions may still opt for active innovation if they are able to impose sufficiently high internal penalties on passive innovation behavior. However, in this scenario, although both parties will choose active innovation, their returns will not exceed the level of returns that would have resulted from both parties choosing passive innovation simultaneously, leading to a suboptimal equilibrium resembling the Prisoner’s Dilemma. The implication for reality is that, when the government does not implement regulatory measures or the regulatory efforts are weak, relying solely on the internal penalty mechanisms between industry stakeholders to constrain passive innovation behavior may result in a ‘Prisoner’s Dilemma’ situation during collaborative innovation in the emergency industry. Furthermore, this is more likely to occur in emergency industries with weak economic attributes than in those with strong economic attributes. This also provides further evidence of the necessity of government regulation in promoting collaborative innovation in emergency industries with weak economic attributes. Interestingly, the amounts of R&D subsidies do not significantly affect the above results. What does matter is the government’s punishment for passive innovation behavior under the R&D subsidy policy. This is because R&D subsidies are typically based on ex-ante commitments, meaning that whether or not emergency enterprises and academic institutions actively engage in collaborative innovation does not affect their eligibility for receiving subsidies. The above results suggest the existence of an incentive misalignment problem in policy design: R&D subsidies fail to effectively translate into innovation motivation, while the penalty mechanism becomes a key variable in regulating the behavior of both parties. To resolve this dilemma, it is necessary to reconsider the design logic of policy tools, strengthen dynamic incentive mechanisms and ex-post evaluation systems to substantially enhance the efficiency of collaborative innovation.

## 4. Simulation analysis

The previous sections provided a theoretical analysis of the innovation behavior of emergency industry entities under government regulation using the concept of evolutionary game theory. Moving forward, this section employs MATLAB R2022a to conduct numerical simulations of the evolutionary process of behavioral strategies among government departments, emergency enterprises—and UR. The simulations offer a more intuitive method to analyze the impact of key parameters on the behavioral strategies of each entity, thereby exploring feasible paths to achieve collaborative innovation in the emergency industry.

In order to ensure that the numerical simulation results have practical significance, especially for parameters related to the characteristics of the emergency industry, we strive to seek evidence as a reference for parameter assignment. The parameter assignments are as follows:

(1) Based on the emergency equipment procurement announcements published on the Chinese Government Procurement Website, the range of government procurement intensity *K* is set to 0 ~ 8.
(2) Referring to the industrial support policy documents of the Chinese central and local governments, the range of R&D subsidy W is set to 0 ~ 4.
(3) Combining actual conditions: Currently, there are 260 million urban households in China, with a smoke alarm installation rate of less than 10%. If the installation rate increases to 30%, the market potential will exceed 30 billion yuan. Additionally, the overall installation rate of household safety emergency products is less than 5%. Based on the current 490 million households in China, the market size of household emergency products in 2023 is estimated to be approximately 13 billion yuan. Therefore, the current level of public emergency awareness is relatively low. Considering the relative parameter values, the initial level of public emergency awareness $e_{0}$ is set to 0.2. At the same time, to simulate the impact of changes in public awareness, the range of public awareness e is set to 0.2 ~ 0.8. Furthermore, based on the potential market size, the range of the parameter *r*, which represents economic attributes, is set to 6 ~ 10. Additionally, since sudden disaster events often have uncertainty and unpredictability, the parameter *u*, which represents the uncertainty of emergency industry returns, is set to 3.
(4) Given the extensive research on industrial collaborative innovation, some more traditional parameters are assigned values by referencing the research of Hou et al. (2023) and Zan et al., (2019) [48,49], including L=1.5,$C_{F}=1.5$, $C_{L}=1.5$, $C_{I}=1.5$,$S_{1}=1.5$, $S_{2}=1$, μ=5. a=0.6, b=0.55.

The assignment of each parameter only represents the relative size between each parameter. And it is also important to note that, in the model simulation analysis section, when simulating the effects of government regulatory measures, other measures are not considered simultaneously to better analyze the impact of a single regulatory measure. i.e., relevant parameters are set to 0. For example, when analyzing the impact of government procurement intensity *K*, the innovation subsidy *W* is set to 0.

(1) **The Impact of the Economic Attributes *r* and the Degree of Market Return Uncertainty *u***

**Fig 2:**
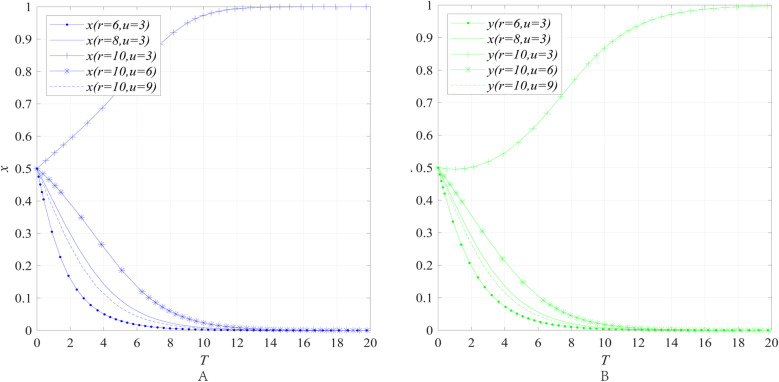
The impact of the economic attributes *r* and the degree of market return uncertainty *u* on the behavioral strategies of entities. **(A)** The impact of the economic attributes *r* and the degree of market return uncertainty *u* on the behavioral strategies of emergency enterprise. **(B)** The impact of the economic attributes *r* and the degree of market return uncertainty *u* on the behavioral strategies of UR.

The impact of the economic attributes *r* and the degree of market return uncertainty *u* on the behavioral strategies of entities can be obtained through numerical simulation, as shown in Fig 2.

Fig 2A and 2B illustrate the impact of the economic attributes *r* and the degree of market return uncertainty *u* on the behavioral strategies of emergency enterprises and UR under the condition where the government does not implement any regulatory measures (i.e., market mechanism), while keeping other parameters constant. The simulation results reveal that when the degree of market return uncertainty *u* is held constant at 3 and the economic attributes *r* increases from 6 to 10, the convergence result of *x* and *y* both changes, shifting from converging to 0 to converging to 1, and when the economic attributes *r* is held constant at 3 and the degree of market return uncertainty *u* increases from 3 to 9, the convergence result of *x* and *y* both changes, shifting from converging to 1 to converging to 0.

The results indicate that the enthusiasm of emergency response enterprises and UR in collaborative innovation is constrained by two factors: first, the relatively weak economic attributes of the emergency industry, and second, the high uncertainty of market returns. These two factors jointly reduce the expected returns of collaborative innovation for both enterprises and UR thereby weakening their motivation to invest in innovation. Specifically, the weak economic attributes of the emergency industry are mainly reflected in its public goods nature and non-market-oriented characteristics. Unlike general industries, some products and services in the emergency industry are often oriented toward meeting societal public safety needs, making it difficult to achieve economic returns through market mechanisms. At the same time, the uncertainty of market returns further exacerbates this issue. Due to the significant fluctuations in market demand within the emergency industry, which is influenced by multiple factors such as policies and disaster frequency, enterprises and UR find it challenging to accurately predict the market prospects of innovation outcomes. This uncertainty further diminishes the expected returns of collaborative innovation for all parties involved, leading to insufficient participation enthusiasm

(2) **The Impact of the Emergency Awareness *e***

**Fig 3:**
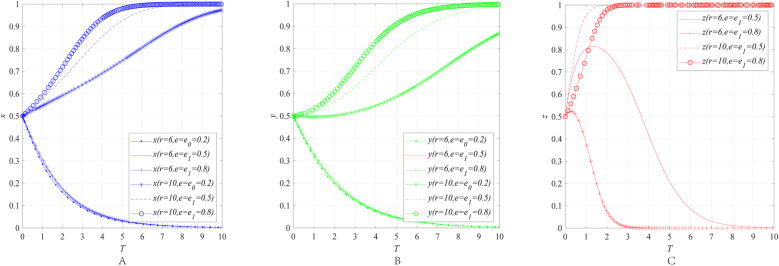
The impact of the emergency awareness *e* on the behavioral strategies of entities. **(A)** The effect of the emergency awareness *e* on the behavioral strategies of emergency enterprise. **(B)** The effect of the emergency awareness *e* on the behavioral strategies of UR. **(C)** The effect of the emergency awareness *e* on the behavioral strategies of government department.

The impact of the emergency awareness *e* on the behavioral strategies of entities can be obtained through numerical simulation, as shown in Fig 3.

Fig 3A–C illustrate the impact of the economic attributes r and the emergency awareness *e* on the behavioral strategies of emergency enterprises, UR and government department. The simulation results reveal that when the economic attributes *r* of the emergency industry remains constant at 6, as public emergency awareness *e* increases from 0.2 to 0.8, *x*, *y* and *z* always converge to 0. Among them, the rate at which *x* and *y* converge to 0 does not change significantly, while the rate at which *z* converges to 0 significantly accelerates. When the economic attributes *r* of the emergency industry remains constant at 10, as public emergency awareness *e* increases from 0.2 to 0.8, the convergence results of *x*, *y*, and *z* all shift from 0 to 1. Moreover, the rate at which *x* and *y* converge to 1 accelerates, while the rate at which *z* converges to 1 slows down.

The results indicate that although enhancing public awareness of emergency preparedness can, to some extent, mitigate the uncertainty of market returns, whether it can effectively incentivize emergency enterprises and UR to participate in collaborative innovation still depends on the economic attributes of the emergency industry. This conclusion aligns with the earlier discussion that the willingness of emergency enterprises and UR to engage in collaborative innovation is constrained by both the economic attributes of the emergency industry and the uncertainty of market returns. Specifically, when the economic attributes of the emergency industry are weak, even if the government takes measures such as promoting emergency knowledge and establishing emergency science education bases to enhance public awareness, thereby stabilizing product demand and returns, the external benefits of collaborative innovation between emergency enterprises and UR remain limited due to the small market size of the products, i.e., weak economic attributes face a “marketization paradox”. As a result, it is difficult to stimulate the collaborative innovation enthusiasm of both parties. At the same time, when government departments implement measures to raise public awareness, they not only incur costs but may also face limited effectiveness, leading to a gradual abandonment of such efforts. However, when the economic attributes of the emergency industry are strong, the improvement of public emergency awareness can significantly enhance the enthusiasm of emergency enterprises and UR for collaborative innovation. An interesting phenomenon is that when the government’s willingness to further enhance public awareness slightly weakens, it does not negatively impact the collaborative innovation enthusiasm of emergency enterprises and UR. This suggests that market-driven interest mechanisms play an important role in promoting collaborative innovation in emergency industries with strong economic attributes.

(3) **The Impact of the R&D Subsidy *W***

**Fig 4:**
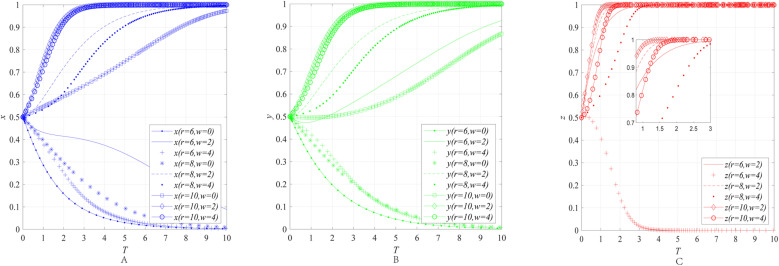
The impact of the R&D subsidy *W* on the behavioral strategies of entities. **(A)** The impact of the R&D subsidy *W* on the behavioral strategies of emergency enterprise. **(B)** The impact of the R&D subsidy *W* on the behavioral strategies of UR. **(C)** The impact of the R&D subsidy *W* on the behavioral strategies of government department.

The impact of the R&D subsidy *W* on the behavioral strategies of entities can be obtained through numerical simulation, as shown in Fig 4.

Fig 4A–C illustrate the impact of the R&D subsidy ***W*** on the behavioral strategies of emergency enterprises, UR and government department. The simulation results reveal that when the economic attributes *r* of the emergency industry remains constant at 6, as the R&D subsidy ***W*** increases from 0 to 2, *x* still converges to 0, while *y* and *z* converge to 1. However, as the R&D subsidy *W* increases from 2 to 4, the rate at which *x* converges to 0 accelerates, and the convergence results of *y* and *z* shift from 1 to 0. When the economic attributes *r* of the emergency industry remains constant at 8, as the R&D subsidy *W* increases from 0 to 2, *x* and *y* initially shift from converging to 0 to converging to 1. Then, as the R&D subsidy *W* increases from 2 to 4, *x* and *y* still converge to 1, but the convergence rate slows down. When the economic attributes of the emergency industry *r* remains constant at either 8 or 10, as the R&D subsidy *W* increases, *z* always converges to 1, but the convergence rate slows down.

The results indicate that the government’s indiscriminate increase in subsidy for collaborative innovation in the emergency industry is not always beneficial and may instead lead to policy effects deviating or even becoming distorted. Specifically, when emergency enterprises and UR are not fully engaged in collaborative innovation, government departments may, in certain cases, fail to promptly suspend the implementation of subsidy policies. This phenomenon reflects certain limitations of the innovation subsidy mechanism based on ex-ante commitment: regardless of whether emergency enterprises and UR actively participate in the collaborative innovation process, the subsidy policy will be executed as planned in the early stages, highlighting the lack of precision in the innovation subsidy policy. Furthermore, when emergency enterprises and UR already exhibit a high level of enthusiasm for collaborative innovation, excessively increasing R&D subsidy may instead weaken their motivation. This phenomenon may be attributed to the following two reasons: First, blindly raising subsidy levels can exacerbate fiscal pressure, making it difficult to sustain the policy in the long term. Second, innovation subsidy policies are typically based on ex-ante commitments, and excessively high subsidies may lead to subsidy dependency among innovation entities, causing them to rely on subsidies rather than market returns generated through innovation activities, thereby undermining their intrinsic motivation to participate in collaborative innovation.

(4) **The Impact of the Punishment Intensity *F***

**Fig 5:**
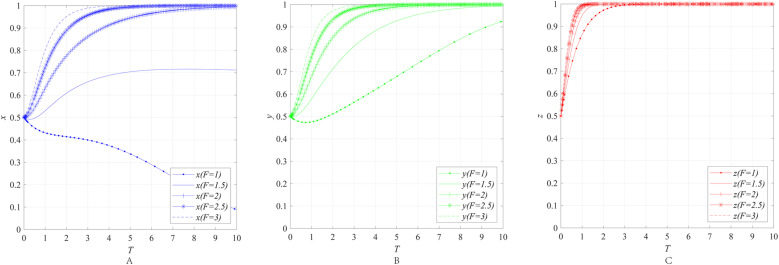
The impact of the punishment intensity *F* on the behavioral strategies of entities. **(A)** The impact of the punishment intensity *F* on the behavioral strategies of enterprise. **(B)** The impact of the punishment intensity *F* on the behavioral strategies of UR. **(C)** The impact of the punishment intensity *F* on the behavioral strategies of government department.

The impact of the punishment intensity *F* on the behavioral strategies of entities can be obtained through numerical simulation, as shown in Fig 5.

Fig 5A– C illustrate the punishment intensity *F* on the behavioral strategies of emergency enterprises, UR and government department. The simulation results reveal that as the punishment intensity *F* increases from 1 to 3, *x* initially shifts from converging to 0 to converging to 1 and the rate of convergence to 1 subsequently accelerates, *y* and *z* always converge to 1, and their convergence rates also increase.

The results indicate that in the process of promoting collaborative innovation in the emergency industry, government departments should consider increasing the penalties for passive innovation behaviors, in addition to implementing incentive measures such as subsidy policies. This can not only help avoid potential deviations and distortions in the effects of subsidy policies but also behaviorally constrain possible passive innovation behaviors by emergency enterprises and UR. Moreover, Proposition 4 in the earlier discussion also points out that appropriately calibrated penalties can help avoid the potential “prisoner’s dilemma” in the collaborative innovation process of the emergency industry. Therefore, this paper argues that government intervention in the collaborative innovation of the emergency industry should strike a balance between the intensity of incentive measures and the intensity of restrictive measures.

(5) The Impact of the Government Procurement Intensity *K*

**Fig 6:**
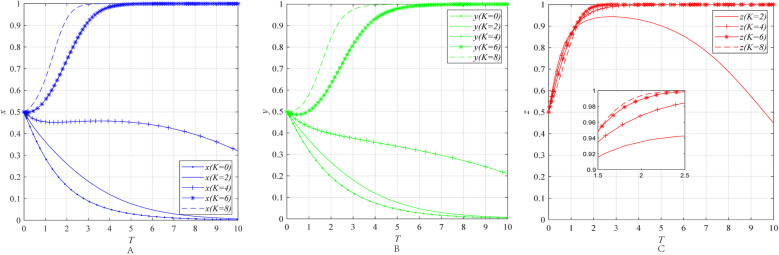
The impact of the government procurement intensity *K* on the behavior strategies of entities. **(A)** The impact of the government procurement intensity *K* on the behavioral strategies of enterprise. **(B)** The impact of the government procurement intensity *K* on the behavioral strategies of UR. **(C)** The impact of the government procurement intensity *K* on the behavioral strategies of government department.

The impact of the government procurement intensity *K* on the behavioral strategies of entities can be obtained through numerical simulation, as shown in Fig 6.

Fig 6A–C illustrate the impact of the government procurement intensity *K* on the behavioral strategies of emergency enterprises, UR and government department. The simulation results reveal that as the government procurement intensity *K* increases from 0 to 8, *x*, *y* and *z* all shift from converging to 0 to converging to 1 gradually.

Compared to the simulation results of the impact of the R&D subsidy *W* on the behavioral strategies of entities, we find that under the same conditions, *x* and *y* are more sensitive to the government procurement intensity *K* than to the R&D subsidy *W.* And the government procurement intensity *K* is more effective in promoting the convergence of *x* and *y* to 1.

The results indicate that government procurement has a significantly positive effect on promoting the participation of emergency enterprises and UR in collaborative innovation, and it is more effective compared to R&D subsidy. The reason is that the government, as a direct source of market demand, can provide stable orders and income sources for emergency enterprises and UR, thereby reducing the uncertainty of market returns. This direct market-pulling effect is more pronounced than that of R&D subsidy. Although R&D subsidy can alleviate some financial pressure, they cannot fully eliminate the risks brought by market uncertainty.

(6) **The Impact of the Government Regulatory Willingness *z***

**Fig 7:**
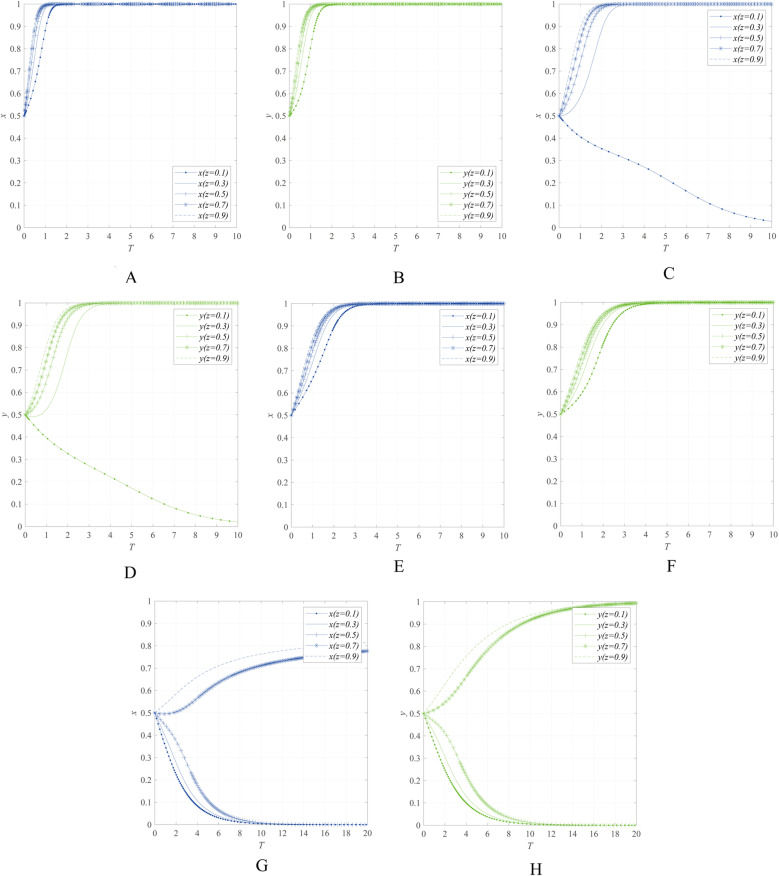
The impact of government regulatory willingness *z* on the behavioral strategies of entities. **(A)** The impact of government regulatory willingness *z* on the behavioral strategies of emergency enterprise when K=8, r=10 and e=0.8. **(B)** The impact of government regulatory willingness *z* on the behavioral strategies of UR when K=8, r=10 and e=0.8.(C) The impact of government regulatory willingness *z* on the behavioral strategies of emergency enterprise when K=8, r=6 and e=0.8. **(D)** The impact of government regulatory willingness *z* on the behavioral strategies of UR when K=8, r=6 and e=0.8. **(E)** The impact of government regulatory willingness *z* on the behavioral strategies of emergency enterprise when W=4, r=10 and e=0.8. **(F)** The impact of government regulatory willingness *z* on the behavioral strategies of UR when W=4, r=10 and e=0.8. **(G)** The impact of government regulatory willingness *z* on the behavioral strategies of emergency enterprise when W=4, r=6 and e=0.8. **(H)** The impact of government regulatory willingness *z* on the behavioral strategies of UR when W=4, r=6 and e=0.8.

The impact of the government regulatory willingness *z* on the behavioral strategies of entities can be obtained through numerical simulation, as shown in Fig 7.

Fig 7A and 7B illustrate when government departments only adopt procurement measures (K=8), the impact of government regulatory willingness *z* on the behavioral strategies of emergency enterprises and UR under the condition of r=10 and e=0.8. Fig 7C and 7D illustrate when government department departments only adopt procurement measures (K=8), the impact of government regulatory willingness z on the behavioral strategies of r=6 and e=0.8. Fig 7E and 7F illustrate when government departments only implement R&D subsidy policy (W=4), the impact of government regulatory willingness *z* on the behavioral strategies of emergency enterprises and UR under the condition of r=10 and e=0.8. Fig 7G and 7H illustrate when government departments only implement R&D subsidy policy (W=4), the impact of government regulatory willingness z on the behavioral strategies of r=6 and e=0.8.The simulation results indicate that regardless of the regulatory measures chosen, when r=10and e=0.8, as the government’s regulatory willingness z decreases from 0.9 to 0.1, *x* and *y* still converge to 1. However, when r=6and e=0.8, *x* and *y* shift from converging to 1 to converging to 0.

The results suggest that when the emergency industry has strong economic attributes and public emergency awareness is at a high level, a decrease in the government’s regulatory willingness will not lead emergency enterprises and UR to choose passive innovation. However, when the emergency industry has weak economic attributes, a decrease in the government’s regulatory willingness will cause emergency enterprises and UR to tend toward passive innovation. This phenomenon reflects the significant differences in the dependence of emergency industries with different economic attributes on government regulatory measures.

Specifically, for emergency industries with strong economic attributes, if government departments focus on enhancing public emergency awareness in the early stages, they can rely more on market mechanisms in the later stages to maintain the active collaborative innovation willingness of emergency enterprises and UR. This indicates that market mechanisms can serve as a viable pathway to promote collaborative innovation in emergency industries with strong economic attributes. For example, taking China’s household safety emergency product market as an example, the current penetration rate of household safety emergency products is less than 5%. Based on the scale of 490 million households in China, the market size of household emergency products in 2023 is approximately 13 billion yuan. If the government effectively enhances public emergency awareness in the future and raises the penetration rate of household safety emergency products to the average level of developed Western countries, the potential market size could exceed 100 billion yuan. This case further demonstrates the important role of market mechanisms in the collaborative innovation of emergency industries with strong economic attributes. Emergency industries with weak economic attributes need to rely on government-led initiatives to promote collaborative innovation. Taking professional fire rescue equipment as an example, such equipment typically lacks mass consumer appeal, and the general public is unlikely to purchase it voluntarily. Therefore, the market demand for these products heavily depends on government procurement and support. According to statistics, in 2023, the number of bidding projects for safety and emergency-related equipment products in China exceeded 40,000, involving a total amount of over 200 billion yuan. Among these, the 528 fire rescue equipment procurement projects recorded on the Chinese Government Procurement Website by national and provincial fire rescue departments alone amounted to more than 4.5 billion yuan. This data fully demonstrates the indispensable role of government departments in the development of emergency industries with weak economic attributes, driving relevant enterprises and universities/research institutions to participate in collaborative innovation through procurement and policy support.

(7) **The Impact of the Benefit Distribution Ratio *a***

**Fig 8:**
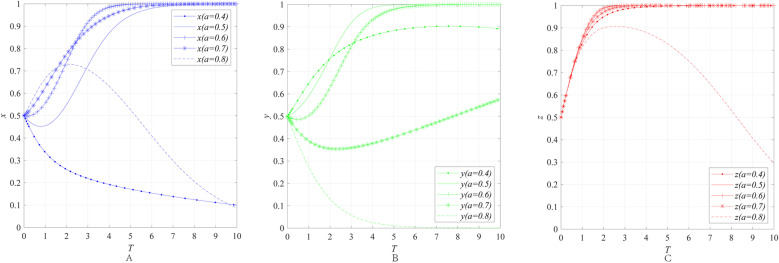
The impact of the impact of benefit distribution ratio *a* on the behavioral strategies of entities. **(A)** The impact of the benefit distribution ratio *a* on the behavioral strategies of emergency enterprise. **(B)** The impact of the benefit distribution ratio *a* on the behavioral strategies of UR. **(C)** The impact of the benefit distribution ratio *a* on the behavioral strategies of government department.

The impact of the benefit distribution ratio *a* on the behavioral strategies of entities can be obtained through numerical simulation, as shown in Fig 8.

Fig 8A–C illustrate the impact of the benefit distribution ratio *a* on the behavioral strategies of emergency enterprises, UR and government department. The simulation results reveal that as the benefit distribution ratio *a* increases from 0.4 to 0.8, the convergence results of *x*, *y* and *z* all change. Specifically, the benefit distribution ratio *a* is too high (low), *x* (*y*) initially shows a tendency to converge to 1but then gradually shifts to converging to 0, and *z* either does not converge to 1, or even if it does converge to 1, the convergence speed slightly decreases compared to when the benefit distribution ratio a is within a certain intermediate range.

The results indicate that these results suggest that establishing a reasonable benefit distribution mechanism is the foundation for ensuring the continuity and effectiveness of collaborative innovation, whether under government regulation or market mechanism. In the process of collaborative innovation, when emergency enterprises or UR account for an excessively high proportion of the benefits, they may initially exhibit high enthusiasm for innovation. However, over time, due to the imbalance in benefit distribution, the other party may gradually lose motivation to participate in collaborative innovation. This imbalance in benefit relationships ultimately leads to the breakdown of collaborative innovation, causing even the emergency enterprises or UR with originally high benefit shares to lose innovation enthusiasm due to the collapse of the cooperative relationship. Furthermore, a reasonable benefit distribution mechanism can not only sustain collaborative innovation between emergency enterprises and UR in the long term but also positively influence the government’s regulatory tendencies. This implies that building a positive and healthy collaborative innovation relationship between emergency enterprises and UR not only enhances the efficiency of collaborative innovation for both parties but also increases the likelihood of obtaining relevant policy support, thereby creating a win-win situation for emergency enterprises, UR, and government departments. The practical implication of this phenomenon is that, in the process of collaborative innovation in the emergency industry, simply increasing benefit distribution to motivate the less enthusiastic party may, in the long run, undermine the stability and sustainability of collaborative innovation. Therefore, when designing benefit distribution mechanisms, it is essential to balance the interests of all parties and avoid damaging long-term cooperative relationships through short-term incentive measures.

## 5. Conclusions and implications

At present, promoting collaborative innovation in the emergency industry is of great significance for enhancing the national emergency response capability and ensuring public safety. In light of this, based on the consideration of relevant characteristic factors of the emergency industry, this study constructed a multi-agent behavioral evolutionary game model, including emergency enterprises, UR, and government departments, to explore the feasible paths of collaborative innovation in the emergency industry. The following conclusions and implications can be drawn from this study.

### 5.1. Conclusions

(1) The economic attributes of the emergency industry and the uncertainty of market returns are key factors that constrain the collaborative innovation and development of the emergency industry. The economic attributes of the emergency industry determine the applicable boundaries of government regulation and market mechanisms in the collaborative innovation process of the emergency industry. Due to the “marketization paradox” in the emergency industry with weak economic attributes, and the fact that emergency enterprises and UR are more likely to face the “prisoner’s dilemma” in the process of collaborative innovation, collaborative innovation via the government regulation-driven pathway is more feasible. As for emergency industries with strong economic attributes, a market mechanism-driven collaborative innovation path becomes feasible under the condition of high public emergency awareness. This is because the uncertainty of market returns in the emergency sector suppresses the enthusiasm of emergency enterprises and UR to participate in collaborative innovation, while the improvement of public awareness can, to some extent, stabilize market returns and reduce investment risks in collaborative innovation. Therefore, the government should strengthen the market-driven revenue mechanism for emergency industries with strong economic attributes by enhancing public emergency awareness, thereby promoting market mechanism-oriented collaborative innovation development. This approach not only helps alleviate the fiscal pressure of government regulation but also enables the government to better concentrate resources and adopt more targeted regulatory measures for collaborative innovation in emergency industries with weak economic attributes. Existing research on industrial collaborative innovation often separately discusses how government regulatory measures or market mechanisms influence the innovation behavior of industrial entities (Hao et al., 2022; Wang et al., 2021; Zhang et al., 2025;) [7,50,51], without integrating government regulation and market mechanisms into a unified framework to address the applicability boundaries of the two in the industrial collaborative innovation process. Rooted in the characteristics of the emergency industry, this study explores this issue and proposes new perspectives.
(2) Regarding the effectiveness of government regulatory measures in promoting collaborative innovation in the emergency industry, we found that government procurement is more effective than subsidy policies. The reason is that while subsidy policies can alleviate some financial pressure, they cannot fully eliminate the risks brought by market uncertainty. Moreover, innovation subsidy policy has a “double-edged sword” effect. Although it can reduce the innovation investment costs for emergency enterprises and UR, thereby playing a positive role in promoting collaborative innovation in the emergency industry under certain conditions, excessively more subsidies can also produce adverse effects, undermining the enthusiasm of emergency enterprises and UR to participate in collaborative innovation. Additionally, increasing the penalties for passive behavior by emergency enterprises and UR in the collaborative innovation process can to some extent avoid the potential effect bias of subsidy policy. This is consistent with the research conclusions of Li ang Gao (2022) [52]. Our study also revealed that government procurement can help address the “prisoner’s dilemma” that may arise during the collaborative innovation process of emergency industries, but innovation subsidy policies have no effect on this. This finding further enriches the understanding of the effects of incentive measures (subsidy policies, government procurement) and punitive measures for behavioral constraints in industrial innovation.
(3) Relying solely on penalty mechanisms among internal industry entities to curb passive innovation behavior, emergency enterprises and UR are likely to fall into a “prisoner’s dilemma” during the collaborative innovation process. Moreover, the weaker the economic attributes of the emergency industry, the higher the risk of facing this dilemma. Establishing a reasonable benefit distribution mechanism can not only stimulate the enthusiasm of emergency enterprises and UR to participate in the collaborative innovation process but also enhance the government’s willingness to adopt regulatory measures, thereby creating a win-win situation for emergency enterprises, UR and government departments. This result indicates that collaborative innovation in the emergency industry is a complex systemic project involving multiple stakeholders, including emergency enterprises, UR and government departments. Not only can external policy environments directly influence the behavior of internal industry entities (emergency enterprises and UR), but the internal relationships among these entities can also indirectly affect the implementation of external policies. Therefore, in promoting collaborative innovation in the emergency industry, it is essential to fully consider the interest relationships among internal industry entities as well as external policy environment factors to achieve sustainable development of collaborative innovation.

### 5.2. Implications

#### 5.2.1. Theoretical implications.

(1) The analysis of micro-level mechanisms provides a new perspective for research on collaborative innovation in the emergency industry. This study, based on evolutionary game theory, reveals the strategic choices and interactions among the government, emergency enterprises, and UR in the collaborative innovation process from a micro-level perspective. This research fills the gap in existing literature on micro-level mechanisms and expands the application of evolutionary game theory in the field of emergency management, providing a new analytical framework and methodological support for future research.
(2) Economic attributes and market uncertainty are key influencing factors for collaborative innovation in the emergency industry. Breaking through the traditional analytical framework of industrial collaborative innovation, this study incorporates factors such as the economic attributes of the emergency industry and market return uncertainty into the model, uncovering the unique pathways of collaborative innovation in the emergency industry. This research not only deepens the understanding of the inherent laws of collaborative innovation in the emergency industry but also enriches the theoretical connotation of industrial collaborative innovation, offering valuable insights for research on collaborative innovation in other industries with special attributes.
(3) The applicability boundaries of government regulation and market mechanisms provide a theoretical basis for policy formulation. The emergency industry encompasses a wide range of sectors, with products exhibiting characteristics of both general goods and public goods, as well as general-purpose and specialized-purpose goods. Formulating industrial policies without classification makes it difficult to achieve precise and targeted industry cultivation. This study proposes feasible pathways for collaborative innovation based on the economic attributes of the emergency industry and discusses the effects of specific regulatory measures (e.g., government procurement, R&D subsidy), providing a theoretical foundation for differentiated policy design.

#### 5.2.2. Managerial implications.

(1) The economic attributes of emergency products are assessed based on factors such as the importance of emergency products, the target of use, and the market demand potential, and they are classified accordingly. For emergency industries with weaker economic attributes, the leading role of the government in collaborative innovation should be further clarified, and the guiding role of government regulation should be strengthened. By formulating emergency product and service demand catalogs and purchasing public emergency products and services openly, a market application space for collaborative innovation results can be provided. For emergency industries that have strong economic attributes but currently have a weak performance, the traction of a large-scale demand market should be valued, and the timing of public resource entry should be decided prudently. For example, household emergency products have great market potential and should be promoted through emergency safety knowledge to further improve public emergency safety awareness and achieve “expansion and quality improvement” of consumption, thereby promoting the transformation of industrial collaborative innovation from government regulation-driven to market mechanism-guided innovation.
(2) Safety and emergency industry conferences should be actively held to encourage localities to hold development conferences and expos for safety and emergency equipment; this would promote the docking of industry and research and the connection of production and demand. Emergency enterprises, research institutions, and universities should be guided to form innovation alliances in the emergency industry and to achieve collaborative innovation through contract research and development, joint problem solving, patent sharing, and other methods. For the internal participants of the innovation alliance, the rationality of cost sharing and benefit distribution should be valued; they can be comprehensively considered based on the resources invested by each party, the risks undertaken, and the intellectual achievements contributed, to ensure that all parties can fairly share the economic benefits brought by the results in the process of collaborative innovation.
(3) In the process of guiding and promoting collaborative innovation by the government, it is indeed necessary to pay attention to both incentive measures (e.g., innovation subsidies) and punitive measures. Such a balanced strategy helps to ensure that all parties are motivated to actively participate and to act in accordance with established rules, thereby promoting the realization of industrial collaborative innovation.

### 5.3. Limitations

Despite the encouraging results, this study also had the following limitations. First, in order to derive clear insights, we assume that collaborative innovation can always lead to more market returns. However, innovation may face failure. Taking the risks into consideration in the future will enrich the results of this study. Second, this study revealed the key factors and intrinsic mechanisms affecting collaborative innovation pathways from theoretical perspective by constructing an evolutionary game model. Collecting research data for empirical study about the collaborative innovation in the emergency industry is of great importance in guiding significant practices.
